# Central-line team effort: Recognizing problematic central-line insertion sites in nursing homes

**DOI:** 10.1017/ash.2023.331

**Published:** 2023-09-29

**Authors:** Kristine Nguyen, Raveena D. Singh, Shruti Gohil, Raheeb Saavedra, John Billimek, Steven Tam, Susan Huang

## Abstract

**Background:** Recognizing problematic central-line insertion sites is an important activity for CNAs, LVNs, and RNs in nursing homes (NHs). Although CNAs are not responsible for assessing central lines, they are often the first line of defense for noticing and relaying problems with a line because of the greater amount of time they spend with residents. We sought to assess how well CNAs, LVNs, and RNs could identify problematic insertion sites in NHs. **Methods:** We conducted a prospective observational study of central-line care in 8 NHs in Orange County, California. A convenience sample of central lines with a range of problematic elements was selected for quality improvement purposes. Research staff used standardized observation forms to evaluate presence of redness, cloudy drainage, and dressing integrity and change date. NH CNAs, LVNs, and RNs were asked to directly observe devices and to comment on problems or concerns. Participants were also asked open-ended questions about elements for a “picture-perfect line” and standard frequency of line checks and dressing changes. Failures to recognize existing problematic elements were tabulated for CNAs and LVNs or RNs separately. **Results:** In total, 50 CNAs (nursing home range, 3–6) and 50 LVNs and RNs (NH range, 4–6) directly observed lines with 131 problematic elements, including redness (N = 36), cloudy drainage (N = 30), peeling dressings (N = 29), and inappropriately dated dressing (N = 36). Failure to identify problematic elements involved redness [CNAs (50%) and LVNs or RNs (53%)], cloudy drainage [CNAs (40%) and LVNs or RNs (39%)], peeling dressings [CNAs (100%) and LVNs or RNs (87%)], and inappropriately dated dressing [CNAs (71%) and LVNs or RNs (68%)]. For both CNAs and LVNs and RNs, recognition of redness and cloudy drainage improved with severity. Failure to recognize minimal erythema [CNAs (83%) and LVNs or RNs (58%)] was higher than substantial erythema [CNAs (54%) and LVNs or RNs (50%)]. Similarly, failure to recognize minimal cloudy drainage [(CNAs (67%) and LVNs or RNs (50%)] was higher than substantial cloudy drainage [CNAs (42%) and LVNs or RNs (36%)]. Overall, identification of problematic elements did not vary by whether the staff member was assigned to care for that resident. Descriptions of “picture-perfect lines” were uniformly poor, with respondents not knowing what elements to mention. **Conclusions:** Failure to recognize redness, cloudy drainage, peeling dressings, and lapses in dressing change dates was common for CNAs and LVNs and RNs in nursing homes. This lack of recognition could prevent proper response to early and late signs of localized infection at central-line sites. Dedicated training regarding key elements of a “picture-perfect line” is needed, including changing the threshold for concern for both small and large amounts of redness and pus.

**Figure:**
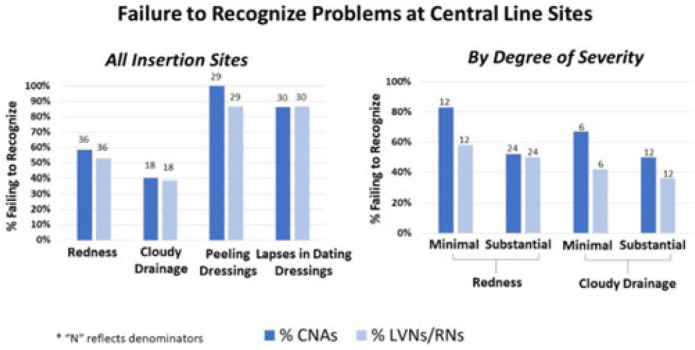

**Disclosures:** None

